# Are greenhouse gas fluxes lower from ley or perennial fallow than from arable organic soils? A systematic review protocol

**DOI:** 10.1186/s13750-023-00310-5

**Published:** 2023-08-25

**Authors:** Alena Holzknecht, Örjan Berglund, Magnus Land, Jacynthe Dessureault-Rompré, Lars Elsgaard, Kristiina Lång

**Affiliations:** 1 Department of Soil and Environment, Swedish University of Agricultural Sciences, Box 7014, 750 07 Uppsala, Sweden; 2 Formas, Box 1206, 111 82 Stockholm, Sweden; 3 Soil and Agri-Food Engineering Department, Faculty of Agriculture and Food Sciences, Laval University, 2325 Rue de L’Université, Québec City, QC G1V 0A6 Canada; 4 Department of Agroecology, Aarhus University, Blichers Allé 20, 8830 Tjele, Denmark; 5 Natural Resources Institute Finland (Luke), Latokartanonkaari 9, 00790 Helsinki, Finland

**Keywords:** Gas fluxes, Climate change, Land-use, Peat soils, Policy

## Abstract

**Background:**

Cultivated peatlands are widespread in temperate and boreal climate zones. For example, in Europe about 15% of the pristine peatland area have been lost through drainage for agricultural use. When drained, these organic soils are a significant source of greenhouse gas (GHG) emissions. To reach climate goals, the agricultural sector must reduce its GHG emissions, and one measure that has been discussed is changing land use from cropland to ley production or perennial green fallow. This management change leads to lower reported emissions, at least when using the IPCC default emission factors (EF) for croplands and grasslands on organic soils (IPCC 2014). However, there was a limited background dataset available for developing the EFs, and other variables than management affect the comparison of the land use options when the data originates from varying sites and years. Thus, the implications for future policies remain uncertain. This protocol describes the methodology to conduct a systematic review to answer the question of whether ley production or perennial green fallow can be suggested as a valid alternative to annual cropping to decrease GHG emissions on organic soils in temperate and boreal climate.

**Methods:**

Publications will be searched in different databases and bibliographies of relevant review articles. The comprehensiveness of the search will be tested through a list of benchmark articles identified by the protocol development team. The screening will be performed at title and abstract level and at full text level, including repeatability tests. Eligible populations are organic agricultural soils in temperate and boreal climate regions. Interventions are grasslands without tillage for at least 3 years, and comparators are annual cropping systems within the same study as the intervention. The outcome must be gas fluxes of either carbon dioxide (CO_2_), nitrous oxide (N_2_O), or methane (CH_4_), or any combination of these gases. Studies will go through critical appraisal, checking for internal and external validity, and finally data extraction. If possible, a meta-analysis about the climate impact of perennial green fallow compared to annual cropping on organic soils will be performed.

**Supplementary Information:**

The online version contains supplementary material available at 10.1186/s13750-023-00310-5.

## Background

About 11% of all global greenhouse gas (GHG) emissions come from agriculture [1]. Therefore, the agricultural sector has a significant role in reaching international and national emission reduction targets, such as the agreement from COP26 in Glasgow 2021 and the Swedish environmental goal "limited climate impact". However, agriculture has a wide set of alternative land uses and management methods, and there is limited knowledge of the net climate impact of the alternatives or combinations of different options. Practical advice for individual farmers is often general and is only sometimes considering local conditions (for example, air permeability, organic matter content and soil type).

Although knowledge of the impact of different land uses and management methods is limited, there is a relatively good understanding of the basic mechanisms for the production and turnover of GHG in soils. Research has shown how the production and consumption of carbon dioxide (CO_2_), methane (CH_4_) and nitrous oxide (N_2_O) in the soil are controlled by microbial processes [2, 3]. Under aerobic conditions, organic material (like peat) breaks down to CO_2_ mediated by microbial respiration processes using oxygen as the terminal electron acceptor. Therefore, organic soils that are drained and exposed to oxygen will be a significant source of CO_2_ [4]. Under waterlogged anoxic conditions, microorganisms break down organic material by anaerobic processes, including methanogenesis and denitrification, which produces CH_4_ and various proportions of N_2_O, respectively [5]. In ecosystems with oxic topsoil, CH_4_ produced in deeper anoxic subsoil may be oxidised to CO_2_ by aerobic methanotrophic bacteria during transport towards the atmosphere [6]. The risk of CH_4_ emissions from agricultural land is therefore associated with high levels of organic matter and water in the soil since both these factors will restrict oxygen availability [7]. Nitrous oxide can be produced under aerobic soil conditions as a side reaction in the oxic transformation of ammonia (NH_3_) to nitrate (NO_3_^−^), but the main risk of N_2_O emission relates to anaerobic denitrification of NO_3_^−^ to gaseous N compounds. In summary, microbial processes control GHG emissions with oxygen availability as a main driver, which is itself restricted by soil water content. However, further drivers of GHG emissions relate to soil physical properties, organic matter content, and access to nutrients. For example, it is a combination of nitrogen fertilisation, plant nitrogen uptake and the conditions for anaerobic environments to form, which determine to what extent the soil becomes a source of nitrous oxide. These factors will be influenced by the land use and management methods that are applied at farm level [8, 9]).

Organic soils spread over almost 500 Mha worldwide, of which around 400 Mha is situated in boreal and temperate regions [10]. The area of peatlands drained for management has been estimated at 43–51 Mha globally [11, 12]. In Europe, about 10% of the former peatland area has been lost through drainage for agriculture, forestry, and peat extraction and about 50% of the current peatland area in the EU is classified as degraded [13]. For example, to increase food security in the beginning of the nineteenth century, the Swedish government started projects to drain peatlands to be able to cultivate them [14]. A side effect was that the drained soils started to release CO_2_ into the atmosphere. Today, 7%, 9%, 10% and 14% of Danish, Swedish, Norwegian, and Finnish agricultural soils respectively are cultivated peatlands [15–18]. Even though the organic soils constitute a relatively small proportion of the arable land, they are considered to be a major source of both CO_2_ and N_2_O [19, 20].

Rewetting drained organic soils is a recognised mitigation tool to reduce GHG emissions [21] and is also supported by the European Union (see *Proposal for a Nature Restoration Law*, European Commission 2022 [22]). However, rewetting arable organic soils would make them largely unsuitable for food production and thus may induce some GHG leakage by shifting the cultivation to other areas. As an alternative, it has been suggested that organic agricultural soils could be used to produce ley or turned into perennial green fallow (Per Bodin, Swedish Board of Agriculture, personal communication, 2022). Some Nordic countries see potential in these interventions, but there is some uncertainty and lacking consensus regarding their effectiveness. Other measures tested in Sweden that allow the cultivation of peat soil at the same time as GHG emissions potentially are reduced, include different grasses [23], the addition of foundry sand [24], the addition of lime [25], different cultivation systems [26], different cultivation intensities [27], raised groundwater table [28] and abandonment [29]. A stakeholder group (representatives from farmers, advisory board, regional government, farmers union and the Swedish Board of Agriculture) gave input and ideas throughout the projects. So far, the only measure that has shown reduced emissions is the sand treatment.

Since there is high political pressure to reduce the GHG emissions from peat soil, and the IPCC emission factors [30] encourage Nordic countries to use ley as a solution, there is a need to strengthen the scientific evidence base on these mitigation measures. Scientific publications and compilations of studies looking into this field are often comparing the treatments without taking into account that the fields for annual and grass crops may originally have been selected, e.g., based on the peat quality. Thus, the compared data does not originate from a homogeneous set of sites or the same years. Other variables than the treatment may have influenced the outcome, such as climate, weather conditions, time and difference in soil properties, or an annual grass crop may have been used in the comparison instead of long-term ley [19, 31–33]. The Swedish Board of Agriculture has expressed a need for a systematic review of existing research results to find out what evidence there is to justify the suggested interventions. The stakeholders mentioned above were invited to a meeting at an early stage in the planning of the forthcoming systematic review, where they were asked to share their thoughts and ideas about the systematic review.

## Objective of the review

The question attempted to be answered in the forthcoming systematic review is: ”What is the effect of ley or perennial green fallow on the flux of greenhouse gases from agricultural organic soils?” The question emerged in a Nordic context, but we will use data from other parts of the world if they meet the eligibility criteria, and we believe that the results and conclusions of the review should be valid also for other countries with similar agricultural practices, in any boreo-temperate climate zone.

The PICO (Population, Intervention, Comparator and Outcome) elements of this question are:

- **Population:** Organic soils on agricultural land in temperate and boreal climate zones. Such organic soils are often drained peatland, but other origins may occur.
- **Intervention:** Using land for grazed or ungrazed, permanent or cultivated grassland (ley) or setting land aside from agricultural production (perennial green fallow) without attempt to raise the groundwater level. Rewetted grasslands are thus not included. Growing woody energy crops is not an eligible intervention or comparator. Growing grass-like energy crops is an eligible intervention.
- **Comparator:** Using land for various crop rotations involving annual crops. Land uses may be categorised regarding tillage, fertilisation, and other management practices.
- **Outcome:** Flux of CO_2_, N_2_O, or CH_4_.

The PICO elements are defined in more detail in the section on study eligibility criteria. It should be noted that issues related to the concept of GHG leakage are outside the scope of this review, although such considerations may influence the overall assessment of land use changes on organic soils.

## Methods

### Searching for articles

No time or document type restrictions will be applied, and publications for which full texts are unavailable will be recorded and reported.

#### Bibliographic databases

The searches in bibliographic databases will be conducted using English search terms, including articles in other languages with English titles and abstracts. The search string comprises three substrings related to the population, intervention, and outcome, respectively (see Table 1). The substrings will be combined with the Boolean operator AND. The format of the search strings will be adapted to each database (see Additional file 1). Searches will be made in the bibliographic databases shown in Table 2. There will be no restrictions regarding publication dates or types.

**Table 1:** Substrings, combined with the Boolean operator AND will be used for searches in bibliographic databases

#	Substring
1	"organic soil" OR "organic soils" OR peat* OR histosol* OR "muck sediment" OR "muck sediments" OR "muck soil" OR "muck soils" OR gyttja OR moorsh* OR wetland* OR turf* OR coprogenous OR muskeg OR suo OR mud OR muds OR swamp OR swamps OR lowland* OR fen OR fens OR mire OR mires OR marsh* OR morass OR quag* OR gley* OR "carbon rich" OR "black soil" OR "black soils" OR bog* OR "high organic carbon" OR hydromorphic
2	grass OR grassland* OR ley* OR fallow OR pasture OR forage OR perennial* OR mesocosm* OR lysimeter* OR semifield* OR legume* OR pulse* OR alfalfa* OR lupin* OR bean* OR lentil* OR clover* OR meadow* OR timothy OR set-aside OR setaside OR pea OR peas OR crop* OR graz*
3	"greenhouse gas" OR "greenhouse gases" OR "carbon dioxide" OR CO2* OR "carbon emission" OR "carbon emissions" OR "nitrous oxide" OR "nitrous oxides" OR N2O OR "laughing gas" OR methane OR CH4 OR "global warming potential" OR GHG* OR "net ecosystem exchange" OR "net ecosystem production" OR respiration OR "carbon balance" OR "trace gas" OR "trace gases" OR NEE OR NEP OR "carbon turnover" OR "eddy covariance" OR "dinitrogen oxide" OR "dinitrogen monoxide" OR "marsh gas"

The asterisk (*) is a wildcard denoting none or any string of characters

**Table 2:** Bibliographic databases that will be used in the literature searches

Database	Search fields	Publisher and URL
Scopus	title, abstract and keywords	Elsevier, https://www.scopus.com/
Web of Science Core Collection^1^	topic	Clarivate Analytics, https://clarivate.com/products/web-of-science/
CAB Abstracts	topic	Clarivate Analytics, https://clarivate.com/products/web-of-science/
ProQuest Natural Science Collection^2^	Abstract and summary text	Proquest, https://www.proquest.com/
Directory of Open Access Journals (DOAJ)	all fields	https://doaj.org/

^1^Including Science Citation Index Expanded™, Social Sciences Citation Index®, Arts & Humanities Citation Index®, Emerging Sources Citation Index, Conference Proceedings Citation Index – Science, Conference Proceedings Citation Index—Social Sciences & Humanities

^2^Including AGRICOLA, Agricultural Science database, Environmental Science database, Environmental Science index, Biological Science database, Biological Science index, Earth, atmosphere & Aquatic Science database

#### Grey literature

Searches for grey literature will be performed using the search engine Google Scholar through Publish or Perish [34]. In these searches, simplified search strings with search terms in English, German, French, Swedish, Finnish, and Danish will be used (see Additional file 1). The first 300 search results for each search string using search terms in English will be screened for relevance, whereas the first 200 search results using the other languages will be screened. Searches for grey literature will also be performed using the archives and databases shown in Table 3.

**Table 3:** Archives and databases to be searched for grey literature

Archive / database	URL	Language
BASE	https://www.base-search.net/	English, German, French, Danish
SwePub	https://swepub.kb.se/	English, Swedish
Finna	https://www.finna.fi/?lng=en-gb	English, Finnish
ProQuest Theses and Dissertations	https://www.proquest.com/	English

#### Supplementary searches

For a number of key search results, we will explore related papers using ResearchRabbit [35]. Further, the protocol development team will contact a list of relevant researchers and other stakeholders, asking for additional literature of interest. For this purpose, a letter template has been written. Also, the bibliography of relevant review articles and meta-analyses will be screened for potentially relevant articles, i.e., “snowballing”. We will also search specialist websites, such as environmental protection agencies or boards of agriculture in countries relevant for the review as defined in the PICO. The websites will be identified in collaboration with stakeholders during the review process and reported in the systematic review.

#### Estimating the comprehensiveness of the search

The comprehensiveness of the search was tested through a list of benchmark articles that the protocol development team identified as relevant for answering the systematic review question (**see **Additional file 2). All but one of the articles indexed in at least one of the searched bibliographic databases were captured by the search strings used. The one missing article [36], in Danish, has a short English abstract with little information. Although relevant to the review question, it does not conform with our inclusion criteria on the outcome. Therefore, we have not judged it meaningful to adjust the search string any further to capture this article. The searches using Google Scholar with search strings in English capture all benchmark publications classified as grey literature except one thesis (Drösler [37]). However, when searching for this publication using the title as the search string, we find at least one web page with this publication and all the words in our Google Scholar search strings. It should thus have been picked up by the searches, but for some reason it was not ranked among the top 300 search results. We judge it unfeasible to adjust the search strategy any further, but it is still possible that this publication will be captured by the searches using search terms in German.

### Article screening and study eligibility criteria

#### Screening process

All studies identified by the above search criteria will be screened to determine inclusion based on the eligibility criteria below. The screening will first be carried out on the title and abstract level and subsequently on the full-text level, deciding for inclusion in the next screening stage in case of uncertainty. The repeatability of the screening process was tested at the abstract stage with 600 publications, which were divided into two groups and screened by three members of the protocol development team in each group. The test articles were retrieved in preliminary searches on Web of Science. After the test screening, the eligibility criteria were discussed among all members of the review team. Having clarified the eligibility criteria, we could resolve the disagreements. The final screening will be divided between two reviewers at the title and abstract level. After double-screening another subset of 300 articles, the consistency between the two reviewers will be reassessed, and if necessary, the eligibility criteria will be clarified again. This procedure will be repeated until we are convinced that the eligibility criteria are interpreted and applied consistently among the two reviewers. At least 10% of the records will be double screened. After that, the screening will continue in single mode. When assessing the consistency between the two reviewers, Kappa tests will be used. However, we will not define any Kappa value a *priori* that must be exceeded. The Kappa values will rather be seen as a support to our assessments and will be reported in the systematic review. At the full-text level, all records will be screened by at least two reviewers. An additional file will provide a list of articles excluded at the full-text stage with reasons for exclusion.

##### Eligibility criteria

The studies will be screened with regard to the population, intervention, comparator, outcome, and study design.

##### Eligible population:

To qualify for this review, the article must include organic soils on agricultural land in temperate and boreal climate zones. As definitions of organic soils vary [38], there will be two categories: “true” peat soils defined as Histosols [39] or having an organic carbon (OC) content > 12% and peat depth > 30 cm, and shallow and/or lower organic carbon peat soils with > 6% OC and > 10 cm depth. The latter may not qualify as peat soils according to many definitions, but with a high bulk density such organic soils nevertheless have the potential for high emissions [38]. The omission of further initial restrictions should prevent the exclusion of relevant data as long as the agricultural system is relevant to the review question. The climate zones considered in this study are Cfb (warm temperate, fully humid, warm summers) and Dfa, Dfb, and Dfc (snow climate, fully humid) according to the Köppen climate classification [40]. As the climate zone is not reported in all studies, and as the classification may have changed over time, the eligibility of all studies will be based on the present classification according to the World Map of the Köppen-Geiger Climate Classification published at https://koeppen-geiger.vu-wien.ac.at/present.htm.

##### Eligible intervention:

To be included, articles must include grazed or ungrazed, permanent or cultivated grassland (ley) or land set aside from agricultural production (perennial green fallow). Ley must be continuous, i.e., without tillage for at least three years. The minimum of three years is somewhat arbitrary. Still, it is reasonable to assume that it will take some time after conversion to grassland before a measurable effect can be detected. Also, a minimum of three years of continuous ley is, e.g., required by the Swedish Board of Agriculture to receive environmental payments [41]. Rewetting peatland is not an eligible intervention. Growing woody energy crops is not an eligible intervention. Growing grass-like energy crops is an eligible intervention, as such may have similar characteristics as other grassland species.

##### Eligible comparator:

Studies that will be included use the land for various crop rotations involving annual crops. We will record the specific crops or crop rotations as potential effect modifiers. Every study needs a ley comparison within the same study, where outcomes (i.e., GHG fluxes) were measured with the same method and in similar peat soil conditions, climate, location, etc., to make them as comparable as possible.

##### Eligible outcome:

For a study to be included, it must report either the flux of CO_2_, N_2_O, CH_4_, or several of those. Gas fluxes must have been measured directly using, for example, dark or transparent chambers, eddy covariance measurements, or concentration gradient methods. Estimations of gas fluxes based on indirect measures, such as soil subsidence or changes in soil organic carbon stocks, are not eligible. The flux of CO_2_ may be reported as net ecosystem exchange (NEE), carbon balance, or soil respiration. As the meaning of these outcome measures differs, we will note which one of them was reported for each study.

##### Eligible study designs and other study characteristics:

We expect that most studies will have a Control-Impact (CI) study design. Still, we will not, by default, exclude any other study design that involves an eligible control, e.g., a Before-After (BA) or a Before-After Control-Impact (BACI) study design. We will not impose initial limits on study *characteristics* like study duration, number of replicates or sampling frequency as we are not expecting many suitable studies. Instead of putting numbers as restrictions, it must be clear that the article describes a system that can answer the review question. Suitability and data quality will rigorously be rated in the study validity assessment. Mesocosm studies are eligible, but the mesocosms should be dimensioned large enough (larger than approximately 0.5 m^2^) and contain soil sufficiently undisturbed to mimic a full-scale grassland. Modelled data will not be included, but studies might be tracked back to check for the input (model validation) data.

### Study validity assessment

Critical appraisal of relevant studies will include an assessment of internal and external validity.

#### Internal validity

The assessment of internal validity will be based on the risk of bias. To assess the risk of bias in individual studies, we will use a modified version of the CEE Critical appraisal tool, version 0.3 [42]. We have chosen to modify the existing tool since we have judged that all criteria and questions within each criterion are not applicable to the planned systematic review. In the modified critical appraisal tool, we consider five criteria (sources of bias). These are confounding biases, selection biases, performance biases, detection biases, and outcome assessment biases. For each source of bias, there is a set of questions which should be answered with “yes”, “no”, or “unclear”. Depending on how the questions are answered, the risk of bias is for each source judged to be “low”, “medium”, “high”, or unclear”. Finally, the overall risk of bias is determined based on the risk of bias associated with each source, according to Table 4. The critical appraisal tool is provided as an Excel file (Additional file 3) and is also illustrated in Additional file 4.

**Table 4:** Criteria for assessing the overall risk of bias for individual studies

Risk of bias level	Explanation
Overall low risk of bias	The study is considered to have low risk of bias for all risk-of-bias criteria for the findings
Overall medium risk of bias	The study is considered to have medium risk of bias in at least one risk-of-bias criterion, but not to have high or unclear risk of bias for any risk-of-bias-criteria for the findings
Overall high risk of bias	The study is considered to have high risk of bias in at least one risk-of-bias criterion for the findings
Overall unclear risk of bias	The study is considered to have unclear risk of bias when the reported information is insufficient to assess the risk of bias for at least one criterion, and the risk is judged to be lower than "high" for all other criteria

#### External validity

The external validity of the studies, i.e., the degree to which the studies are appropriate or applicable for answering the review question in a particular context, is primarily assessed during study eligibility screening. Since we will compare different cropping systems, it is important that the crops being cultivated in the studies are relevant to the stakeholders, and possible crops to grow are governed mainly by the climate and soil properties. Thus, climate and soil properties will be fundamental when assessing the external validity of the crops being grown. No study will be excluded based on these factors as long as they are judged to comply with the eligibility criteria. Still, we will record them to assess the strength of evidence for different contexts and conditions. Another aspect of external validity that needs to be accounted for when evaluating the strength of evidence is the transferability of study results from small-scale experimental studies (e.g., mesocosms) to actual farming practices. Therefore, we will also record the type and scale of included studies.

#### Coding for study validity assessment

Critical appraisal and coding for internal study validity will be carried out by four reviewers, and each study will be critically appraised independently by two reviewers. The reviewers will not be allowed to assess the validity of their own work. Disagreements between reviewers will be recorded and reconciled through discussions, seeking to reach a consensus among all reviewers. Metadata needed for the assessment of external validity will be extracted and recorded by two reviewers. To check the consistency between the two reviewers, a subset of studies will be extracted by both reviewers. After the completion of metadata extraction, the two reviewers will check each other’s extractions. If quantitative synthesis is feasible, a sensitivity analysis may be performed, comparing results with and without excluding studies with low validity.

### Data coding and extraction strategy

The articles included for data extraction will be split into two batches, and two reviewers will extract data from one batch each. To check consistency between the reviewers and to detect any mistakes, all articles extracted by one reviewer will be double-checked by the other reviewer. In case of disagreements, consensus will be reached through discussions with the broader review team. Data will be recorded as reported in each study. If necessary and feasible, data will be standardised (e.g., conversion of units) at the analysis stage to allow for direct comparison among studies.

Quantitative data and meta-data about the experimental setup and the greenhouse gas emissions will be extracted into a spreadsheet as in Additional file 5, which will be fully available as additional files in the final systematic review. Outcome data will be recorded in separate Excel files for each article. If repeated measurements have been carried out, the data for all reported time points will be recorded. In cases where outcome data were reported in graphic figures, we will use WebPlotDigitizer [43] to extract data. All outcome data used in the meta-analysis will be available in an Additional file.

### Potential effect modifiers/reasons for heterogeneity

The meta-data to be extracted from studies includes data regarding key sources of heterogeneity. Such potential variables were agreed on in consultation with the protocol development team and can be found in Additional file 5.

The main reasons for heterogeneity in the presented question for both the intervention and the comparator may be different soil parameters like OC content, moisture, pH, bulk density, degree of decomposition or peat depth, as they mutually influence each other, as well as microbial activity and thus GHG emissions [44]. Further, drainage or groundwater table depth, time since drainage, time since conversion to annual cropland and ley/ perennial fallow, tillage practices, and applied fertilisers and crop residues may affect emissions and will be recorded. Finally, measurement methodologies will be reported to account for differences between studies, although data synthesis will rely on relative differences between intervention and comparator per study.

### Data synthesis and presentation

All included studies will be presented in narrative synthesis tables, including the extracted metadata and risk of bias assessments. The quantitative synthesis will be carried out through meta-analysis using a random-effects model. Measurement methods of GHG emissions are diverse and may not be comparable in absolute numbers between studies. As the review question asks for a relative comparison between land uses, the collected data will be analysed by calculating relative differences between intervention and comparator per study. We believe the most suitable effect size for this purpose is the log response ratio (ln R). However, we expect that the included studies will generally have a small number of replicates and that the number of studies in each meta-analysis will be relatively small. Therefore, once the data is extracted, we will test the suitability of ln R using the diagnostic test suggested by Hedges et al. [45] and Lajeunesse [46]. Alternatively, standardised mean difference will be used as effect size.

The degree of heterogeneity between study results will be assessed using the I^2^ statistic. Possible reasons for heterogeneity will be explored through subgroup analyses where, for example, “true” peat soils and lower-carbon organic soils, as defined in the *Eligible population* paragraph, are compared, as well as mesocosm, incubation experiments, and large lysimeters vs field sites. However, we leave the option open to include the mesocosm experiment in the analysis of field sites in case there will not be enough eligible studies. Provided that sufficient studies are included in the meta-analysis, we will construct funnel plots [47] to assess the risk of publication bias. Meta-analyses will be conducted in R using the Metafor package [48]. Results will be visualised through forest plots and presented in tables.

When summary treatment effects (point estimates and confidence intervals) have been estimated, we will explore the possibility of grading the evidence and expressing our confidence in the estimated treatment effects. When grading the evidence, we will consider the internal and external validity of included studies, the number of included studies, context dependency, and the risk of publication bias.

## Supplementary Information

**Additional file 1. **Literature searches.

**Additional file 2. **Benchmark articles that should be captured by the searches.

**Additional file 3. **Critical appraisal tool.

**Additional file 4. **Critical appraisal tool visualisation.

**Additional file 5. ** Metadata extraction sheet.

**Additional file 6. **ROSES form for systematic review protocols.

## Data Availability

Not applicable.

## References

[1] Tubiello FN, Salvatore M, Ferrara AF, House J, Federici S, Rossi S, Biancalani R, Golec RDC, Jacobs H, Flammini A, et al. The contribution of agriculture, forestry and other land use activities to global warming, 1990–2012. Glob Change Biol. 2015;21(7):2655–60. 10.1111/gcb.12865 25580828

[2] Ronn R, Griffiths BS, Ekelund F, Christensen S. Spatial distribution and successional pattern of microbial activity and micro-faunal populations on decomposing barley roots. J Appl Ecol. 1996;33(4):662–72. 10.2307/2404938

[3] Christensen S, Bjornlund L, Vestergard M. Decomposer biomass in the rhizosphere to assess rhizodeposition. Oikos. 2007;116(1):65–74. 10.1111/j.2006.0030-1299.15178.x

[4] Ballantyne DM, Hribljan JA, Pypker TG, Chimner RA. Long-term water table manipulations alter peatland gaseous carbon fluxes in Northern Michigan. Wetl Ecol Manag. 2014;22(1):35–47. 10.1007/s11273-013-9320-8

[5] Ussiri D, Lal R. The role of nitrous oxide on climate change. In: Soil emission of nitrous oxide and its mitigation. Dordrecht: Springer; 2013.

[6] Topp E, Pattey E. Soils as sources and sinks for atmospheric methane. Can J Soil Sci. 1997;77(2):167–78. 10.4141/S96-107

[7] Kasimir Klemedtsson Å, Weslien P, Klemedtsson L. Methane and nitrous oxide fluxes from a farmed swedish histosol. Eur J Soil Sci. 2009;60(3):321–31. 10.1111/j.1365-2389.2009.01124.x

[8] Bardgett R. The Biology of Soil: A community and ecosystem approach. Oxford: Oxford University Press; 2005.

[9] Christensen S, Degorska A, Prieme A. Combined assessment of methane oxidation and nitrification: an indicator of air-borne soil pollution? Biol Fertil Soils. 2001;34(5):325–33. 10.1007/s003740100415

[10] Paul S, Leigeld J. Management of organic soils to reduce soil organic carbon losses. In: Rumpel C, editor. Understanding and fostering soil carbon sequestration. Cambridge: Burleigh Dodds Science Publishing; 2022.

[11] Joosten H. The Global Peatland CO2 Picture: Peatland status and drainage related emissions in all countries of the world: Wetlands International. 2010. https://www.wetlands.org/publications/the-global-peatland-co2-picture/. Accessed 7 Aug 2023.

[12] Leifeld J, Menichetti L. The underappreciated potential of peatlands in global climate change mitigation strategies. Nat Commun. 2018;9:7. 29540695 10.1038/s41467-018-03406-6 PMC5851997

[13] UNEP: Global Peatlands Assessment: The State of the World’s Peatlands, Main Report. Nairobi: United Nations Environment Programme; 2022.

[14] Berglund K: Torvmarken, en resurs i jordbruket igår, idag och även imorgon? In: Svensk mosskultur - Odling, torvanvändning och landskapets förändring 1750-2000. Edited by Runefelt L. Stockholm: Kungl. Skogs- och lantbruksakademien; 2008. In Swedish.

[15] Berglund O, Berglund K. Distribution and cultivation intensity of agricultural peat and gyttja soils in Sweden and estimation of greenhouse gas emissions from cultivated peat soils. Geoderma. 2010;154(3–4):173–80. 10.1016/j.geoderma.2008.11.035

[16] Myllys M, Sinkkonen M. The area and distribution of cultivated organic soils in Finland. Suo. 2004;55:53–60.

[17] Grønlund A, Sveistrup TE, Søvik AK, Rasse DP, Kløve B. Degradation of cultivated peat soils in northern norway based on field scale CO2, N2O and CH4 emission measurements. Arch Agron Soil Sci. 2006;52(2):149–59. 10.1080/03650340600581968

[18] Balslev Greve M, Peng Y, Faurholt Pedersen B, Møller AB, Lærke PE, Elsgaard L, Duus Børgesen C, Leth Bak J, Aagaard Axelsen J, Gyldenkærne S et al: Vidensyntese om kulstofrig lavbundsjord. In: Rådgivningsrapport fra DCA – National Center for Fødevarer og Jordbrug. Edited by Greve MH: Landbrugsstyrelsen, FVM; 2021. https://vbn.aau.dk/da/publications/vidensyntese-om-kulstofrig-lavbundsjord-r%C3%A5dgivningsrapport-fra-dc. Accessed 5 Apr 2023.

[19] Kasimir-Klemedtsson A, Klemedtsson L, Berglund K, Martikainen P, Silvola J, Oenema O. Greenhouse gas emissions from farmed organic soils: a review. Soil Use Manage. 1997;13(4):245–50. 10.1111/j.1475-2743.1997.tb00595.x

[20] Maljanen M, Sigurdsson BD, Gudmundsson J, Oskarsson H, Huttunen JT, Martikainen PJ. Greenhouse gas balances of managed peatlands in the Nordic countries - present knowledge and gaps. Biogeosciences. 2010;7(9):2711–38. 10.5194/bg-7-2711-2010

[21] Wilson D, Blain D, Couwenberg J, Evans CD, Murdiyarso D, Page SE, Renou-Wilson F, Rieley JO, Sirin A, Strack M, et al. Greenhouse gas emission factors associated with rewetting of organic soils. Mires Peat. 2016;17:28.

[22] Directorate-General for Environment: Proposal for a Regulation of the European Parliament and of the Council on nature restoration. COM/2022/304 final. Brussels: European Commission. 2022. https://environment.ec.europa.eu/publications/nature-restoration-law_en. Accessed 18 Aug 2022.

[23] Berglund O, Berglund K, Jordan S, Norberg L. Carbon capture efficiency, yield, nutrient uptake and trafficability of different grass species on a cultivated peat soil. CATENA. 2019;173:175–82. 10.1016/j.catena.2018.10.007

[24] Berglund Ö, Jordan S, Hesse K, Berglund K: Effects of foundry sand addition on yield, penetration resistance and CO2 emission from an agricultural peat soil. In: Geophysical Research Abstracts. Vienna, Austria: EGU General Assembly 2019; 2019. p. 21.

[25] Berglund Ö, Berglund K: Mitigating agricultural greenhouse gas emissions by improved pH management of soils (MAGGE-pH) - Swedish case studies. In: EuroSoil 2021 (virtual): 210823-210827. Geneva, Switzerland: Frontiers Media SA. 2021. https://res.slu.se/id/publ/115369. Accessed 18 Aug 2023.

[26] Norberg L, Berglund O, Berglund K. Seasonal CO2 emission under different cropping systems on Histosols in southern Sweden. Geoderma Reg. 2016;7(3):338–45. 10.1016/j.geodrs.2016.06.005

[27] Berglund Ö, Berglund K: Continuous measurements of CO2 emission from cultivated peat soil - effect of tillage intensity. In: Geophysical Research Abstracts. Vienna, Austria: EGU General Assembly; 2014. p. 16.

[28] Berglund O, Berglund K. Influence of water table level and soil properties on emissions of greenhouse gases from cultivated peat soil. Soil Biol Biochem. 2011;43(5):923–31. 10.1016/j.soilbio.2011.01.002

[29] Berglund O, Katterer T, Meurer KHE. Emissions of CO2, N2O and CH4 from cultivated and set aside drained Peatland in Central Sweden. Front Environ Sci. 2021;9:12. 10.3389/fenvs.2021.630721

[30] Hiraishi T, Krug T, Tanabe K, Srivastava N, Baasansuren J, Fukuda M, Troxler TG, editors. IPCC: 2013 Supplement to the 2006 IPCC Guidelines for National Greenhouse Gas Inventories: Wetlands. Switzerland: IPCC; 2014.

[31] Maljanen M, Martikainen PJ, Walden J, Silvola J. CO2 exchange in an organic field growing barley or grass in eastern Finland. Glob Change Biol. 2001;7(6):679–92. 10.1111/j.1365-2486.2001.00437.x

[32] Lohila A, Aurela M, Tuovinen JP, Laurila T. Annual CO2 exchange of a peat field growing spring barley or perennial forage grass. J Geophys Res-Atmos. 2004;109(D18):13. 10.1029/2004JD004715

[33] Beetz S, Liebersbach H, Glatzel S, Jurasinski G, Buczko U, Hoper H. Effects of land use intensity on the full greenhouse gas balance in an Atlantic peat bog. Biogeosciences. 2013;10(2):1067–82. 10.5194/bg-10-1067-2013

[34] Harzing, A.W: Publish or Perish. 2007. https://harzing.com/resources/publish-or-perish. Accessed 4 Aug 2003.

[35] ResearchRabbit. 2023. https://www.researchrabbit.ai/. Accessed 7 Aug 2023.

[36] Pedersen EF. Compression and mineralization of the peat layer in the Store Vildmose bog Toervelagets sammensynkning og mineralisering i Store Vildmose. Tidsskrift for Planteavl. 1978;82(4):509–20.

[37] Drösler M. Trace gas exchange and climatic relevance of bog ecosystems, Southern Germany. Munich: Technischen Universität München; 2005.

[38] Lourenco M, Fitchett JM, Woodborne S. Peat definitions: a critical review. Prog Phys Geogr. 2023;4(7):506–20. 10.1177/03091333221118353

[39] IUSS Working Group WRB: World Reference Base for Soil Resources. International soil classification system for naming soils and creating legends for soil maps, 4 edn. Vienna, Austria: International Union of Soil Sciences (IUSS); 2022.

[40] Kottek M, Grieser J, Beck C, Rudolf B, Rubel F. World map of the Koppen-Geiger climate classification updated. Meteorol Z. 2006;15(3):259–63. 10.1127/0941-2948/2006/0130

[41] Swedish Board of Agriculture: Miljöersättning för vallodling 2023 (in Swedish). https://jordbruksverket.se/stod/jordbruk-tradgard-och-rennaring/jordbruksmark/vallodling. Accessed 5 Apr 2023.

[42] Konno K, Livoreil B, Pullin AS: Collaboration for Environmental Evidence Critical Appraisal Tool Version 0.3 (Prototype). Collaboration for Environmental Evidence. 2021. https://environmentalevidence.org/cee-critical-appraisal-tool/. Accessed 5 Apr 2023.

[43] Webplotdigitizer: Version 4.6. 2022. https://automeris.io/WebPlotDigitizer/. Accessed 21 Aug 2023.

[44] Boelter DH. Physical properties of peats as related to degree of decomposition. Soil Sci Soc Am J. 1969;33(4):606–9. 10.2136/sssaj1969.03615995003300040033x

[45] Hedges LV, Gurevitch J, Curtis PS. The meta-analysis of response ratios in experimental ecology. Ecology. 1999;80(4):1150–6. 10.1890/0012-9658(1999)080[1150:TMAORR]2.0.CO;2

[46] Lajeunesse MJ. Bias and correction for the log response ratio in ecological meta-analysis. Ecology. 2015;96(8):2056–63. 26405731 10.1890/14-2402.1

[47] Sterne JAC, Egger M. Funnel plots for detecting bias in meta-analysis: guidelines on choice of axis. J Clin Epidemiol. 2001;54(10):1046–55. 11576817 10.1016/S0895-4356(01)00377-8

[48] Viechtbauer W. Conducting meta-analyses in R with the metafor package. J Stat Softw. 2010;36(3):1–48. 10.18637/jss.v036.i03

