# Supplementary material for: Are greenhouse gas fluxes lower from ley or perennial fallow than from arable organic soils? A systematic review protocol
Source: Environ Evid. 2023 Aug 25;12:17. doi: 10.1186/s13750-023-00310-5 (PMC11378765; doi:10.1186/s13750-023-00310-5)
Supplement: Supplementary file 1 — Additional file 1. Literature searches. [file 13750_2023_310_MOESM1_ESM.pdf]

Table 1. Search strings in individual bibliographic databases. Numbers in the left column refer to number of search results obtained in preliminary searches.

Database	Search string
Web of Science<br><br>2725	**(**“organic soil” OR “organic soils” OR peatland OR peatlands OR histosol* OR “muck sediment” OR “muck sediments” OR “muck soil” OR ”muck soils” OR gyttja OR moorsh* OR wetland* OR turf* OR coprogenous OR muskeg OR suo OR mud OR muds OR swamp OR swamps OR lowland* OR fen* OR mire OR mires OR marsh* OR morass OR quag* OR gley* OR “carbon rich” OR “black soil” OR “black soils” OR bog* OR “high organic carbon” OR hydromorphic* **) AND (**grass OR grassland* OR ley* OR fallow OR pasture OR forage OR perennial* OR grazing OR mesocosm* OR lysimeter* OR semifield* OR legume* OR pulse* OR alfalfa* OR lupin* OR bean* OR lentil* OR clover* OR meadow* OR timothy OR set-aside OR setaside OR “pea” OR “peas”**) AND (**greenhouse gas OR “greenhouse gases” OR “carbon dioxide” CO2* OR “carbon emission” OR “carbon emissions” OR “nitrous oxide” OR “nitrous oxides” OR N20 OR “laughing gas” OR methane OR CH4 OR “global warming potential” OR ghg* OR “net ecosystem exchange” OR “net ecosystem production” OR respiration OR “carbon balance” OR “trace gas” OR “trace gases” OR nee OR nep OR “carbon turnover” OR “eddy covariance”**)**
Scopus<br><br>1450	**(**{organic soil} OR {organic soils} OR peatland OR peatlands OR histosol* OR {muck sediment} OR {muck sediments} OR {muck soil} OR {muck soils} OR gyttja OR moorsh* OR wetland* OR turf* OR coprogenous OR muskeg OR suo OR mud OR muds OR swamp OR swamps OR lowland* OR fen* OR mire OR mires OR marsh* OR morass OR quag* OR gley* OR {carbon rich} OR {black soil} OR {black soils} OR bog* OR {high organic carbon} OR hydromorphic***) AND (**grass OR grassland* OR ley* OR fallow OR pasture OR forage OR perennial* OR grazing OR mesocosm* OR lysimeter* OR semifield* OR legume* OR pulse* OR alfalfa* OR lupin* OR bean* OR lentil* OR clover* OR meadow* OR timothy OR set-aside OR setaside OR {pea} OR {peas}**) AND (**{greenhouse gas} OR {greenhouse gases} OR {carbon dioxide} CO2* OR {carbon emission} OR {carbon emissions} OR {nitrous oxide} OR {nitrous oxides} OR N20 OR {laughing gas} OR methane OR CH4 OR {global warming potential} OR ghg* OR {net ecosystem exchange} OR {net ecosystem production} OR respiration OR {carbon balance} OR {trace gas} OR {trace gases} OR nee OR nep OR {carbon turnover} OR {eddy covariance}**)**
CAB Abstracts<br><br>2716	Same as Web of Science
Proquest<br><br>1584	Same as Web of Science
DOAJ[1)]<br><br>312	https://doaj.org/api/v3/search/articles/("organic soil" OR "organic soils" OR peat OR histosol OR "muck sediment" OR "muck sediments" OR "muck soil" OR "muck soils" OR gyttja OR moorsh OR wetland OR turf OR coprogenous OR muskeg OR suo OR mud OR muds OR swamp OR swamps OR lowland OR fen OR fens OR mire OR mires OR marsh OR morass OR quag OR gley OR "carbon rich" OR "black soil" OR "black soils" OR bog OR "high organic carbon" OR hydromorphic) **AND** (grass OR grassland OR ley OR fallow OR pasture OR forage OR perennial OR mesocosm OR lysimeter OR semifield OR legume OR pulse OR alfalfa OR lupin OR bean OR lentil OR clover OR meadow OR timothy OR set-aside OR setaside OR pea OR peas OR crop OR graz) **AND** ("greenhouse gas" OR "greenhouse gases" OR "carbon dioxide" OR CO2 OR "carbon emission" OR "carbon emissions" OR "nitrous oxide" OR "nitrous oxides" OR N2O OR "laughing gas" OR methane OR CH4 OR "global warming potential" OR GHG OR "net ecosystem exchange" OR "net ecosystem production" OR respiration OR "carbon balance" OR "trace gas" OR "trace gases" OR NEE OR NEP OR "carbon turnover" OR "eddy covariance" OR "dinitrogen oxide" OR "dinitrogen monoxide" OR "marsh gas")

[1)] Wildcards are not allowed. The search is conducted using an Application Programming Interface (API) request rather than the search web page.

Table 2. Search strings in English used for searches in Google Scholar. The first 300 records retrieved by each search string will be screened for relevance. Terms in red text indicate a difference compared to the search string in the line above.

No.	Search string
1	cultivated **AND** peat **AND (**“greenhouse gas” OR “greenhouse gases” OR “carbon dioxide” OR CO2 OR “nitrous oxide” OR N2O OR methane OR CH4**)**
2	arable **AND** peat **AND (**“greenhouse gas” OR “greenhouse gases” OR “carbon dioxide” OR CO2 OR “nitrous oxide” OR N2O OR methane OR CH4**)**
3	crop **AND** peat **AND (**“greenhouse gas” OR “greenhouse gases” OR “carbon dioxide” OR CO2 OR “nitrous oxide” OR N2O OR methane OR CH4)
4	cultivated **AND** “organic soil” **AND (**“greenhouse gas” OR “greenhouse gases” OR “carbon dioxide” OR CO2 OR “nitrous oxide” OR N2O OR methane OR CH4**)**
5	arable **AND** “organic soil” **AND (**“greenhouse gas” OR “greenhouse gases” OR “carbon dioxide” OR CO2 OR “nitrous oxide” OR N2O OR methane OR CH4**)**
6	crop **AND** “organic soil” **AND (**“greenhouse gas” OR “greenhouse gases” OR “carbon dioxide” OR CO2 OR “nitrous oxide” OR N2O OR methane OR CH4**)**

Table 3. Possible search terms in non-English languages.

Language	Terms for organic soils	Terms for land use	Terms for greenhouse gases
Danish	Tørv, organisk jord, humus jord, mosejord, lavbundsjord, muldjord, dynd, lavmose, højmose	omdrift, permanent græs, sædskifte, græsmark, fælled, overdrev, opdyrket, græsningsareal, kornmark	kuldioxid, lattergas, metan, sumpgas, kultveilte, klimagasser
German	organischer Boden, organische Substanz, Humus, Humusboden, *moor, *moortorf, Brauntorf, Schwarztorf, Torffeld, Gartentorf, Sumpf, Moor, Torfmoos, Histosol	Grasland, Grünland, Weide*, Alm, Wiese, Dauergrünland, Rasen	Treibhausgas, Klimagas, Kohlenstoffdioxid, Methan, Lachgas
Finnish	turvemaa, orgaaninen maa, eloperäinen maa, lieju, turvepelto, eloperäinen peltomaa	maankäyttö, nurmi, vilja, pelto	kasvihuonekaasu, hiilidioksidi, metaani, dityppioksidi, ilokaasu
French	sol organique, tourbière agricole, tourbière cultivée, histosol	prairie, graminées annuelles, graminées vivaces, cultures annuelles, cultures vivaces	gaz à effet de serre, émissions, émissions de gaz à effet de serre, Dioxide de carbone, méthane, oxyde nitreux, protoxyde de diazote, respiration, respiration du sol
Swedish	torv, kärrtorv, mosstorv, mulljord, mull, gyttja, gyttjejord, myr, svartjord, organogen jord, bleke, kalkgyttja, dy, svartmocka	Vall, bete, betesvall, slåttervall, naturbete, träda, svartträda, övergiven, öppen odling, stråsäd, oljeväxter, spannmålsodling, rotfrukter, potatis, grönsaksodling, energiskogsodling	koldioxid, lustgas, metan, dikväveoxid, växthusgaser
