# Supplementary material for: Are greenhouse gas fluxes lower from ley or perennial fallow than from arable organic soils? A systematic review protocol
Source: Environ Evid. 2023 Aug 25;12:17. doi: 10.1186/s13750-023-00310-5 (PMC11378765; doi:10.1186/s13750-023-00310-5)

Potential sources of bias

Criterion 1.
Risk of confounding biases

1.1. Are there any confounding variables that affect the effectiveness of the intervention (= ley or fallow) on the emissions of greenhouse gas emissions?

1.2. Did the author(s) control for all the potential confounders?

1.3. Is there any justifiable reason for not controlling for all the potential confounders (so that omission of some of the potential confounders is unlikely to influence the assessment of the effectiveness or impact)?

1.4. Were the potential confounders, that were controlled for, (and/or the instrumental variable used if applicable) likely to be measured accurately and precisely enough?

1.5. Did the author(s) analyse the effect appropriately by taking into account the potential confounders, as well as the issue of accuracy and precision of the measurements of the potential confounders (and the instrumental variable if applicable)?

RISK OF BIAS

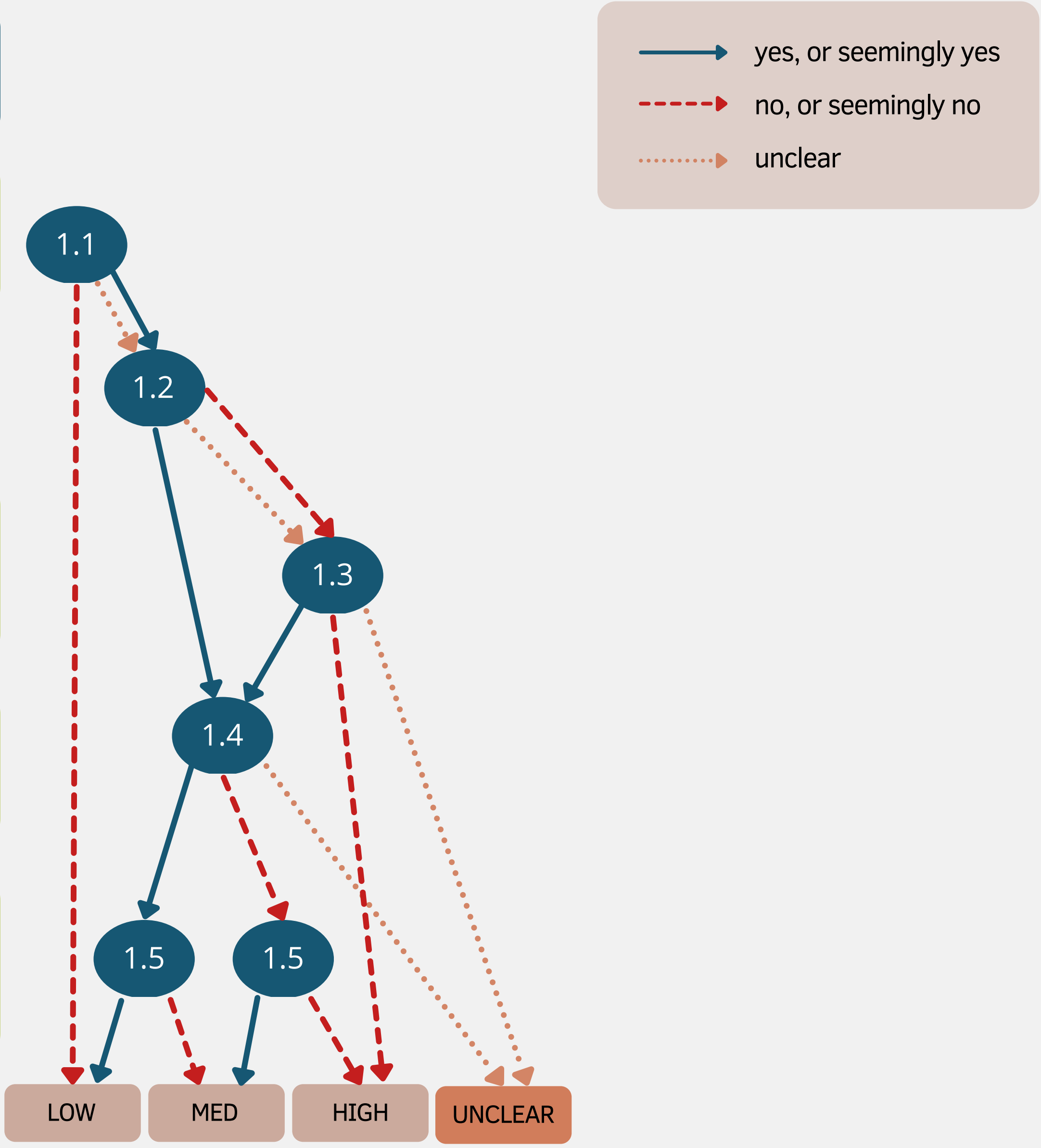

Criterion 2.
Risk of post-intervention/exposure selection biases

2.1. Was the selection of sampling areas after intervention random or systematic?

2.2. Was data from some sampling areas excluded or lost?

2.3. Were the areas included in the study comparable between groups (of land use) and so they allowed a valid comparison to be made?

2.4. Were the differences between groups likely to be explained by the intervention or a variable influenced by the intervention?

2.5. Did the authors adjust for the potential post-intervention/exposure selection bias in an appropriate way?

RISK OF BIAS

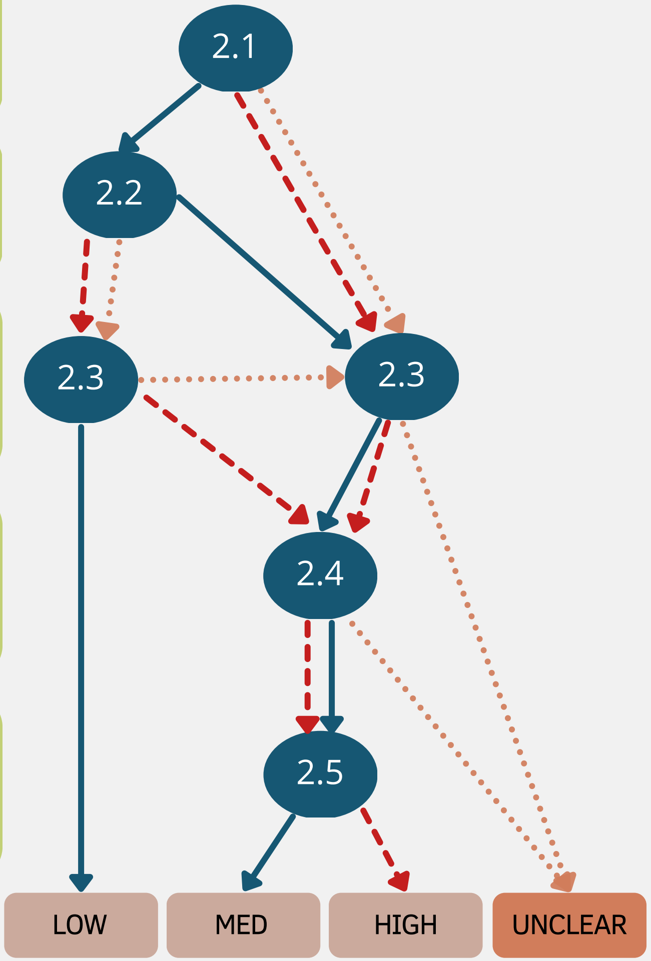

Criterion 3.
Risk of perormance biases

3.1 Were there any temporal alterations of intervention or control treatments that might have an impact on the effectiveness of the intervention?

3.2 Were these deviated treatments unbalanced between intervention and control groups?

3.3. Was the intervention and control, respectively, spatially consistent across all sampling areas?

3.4 Was the uneven intervention considered in analyses?

RISK OF BIAS

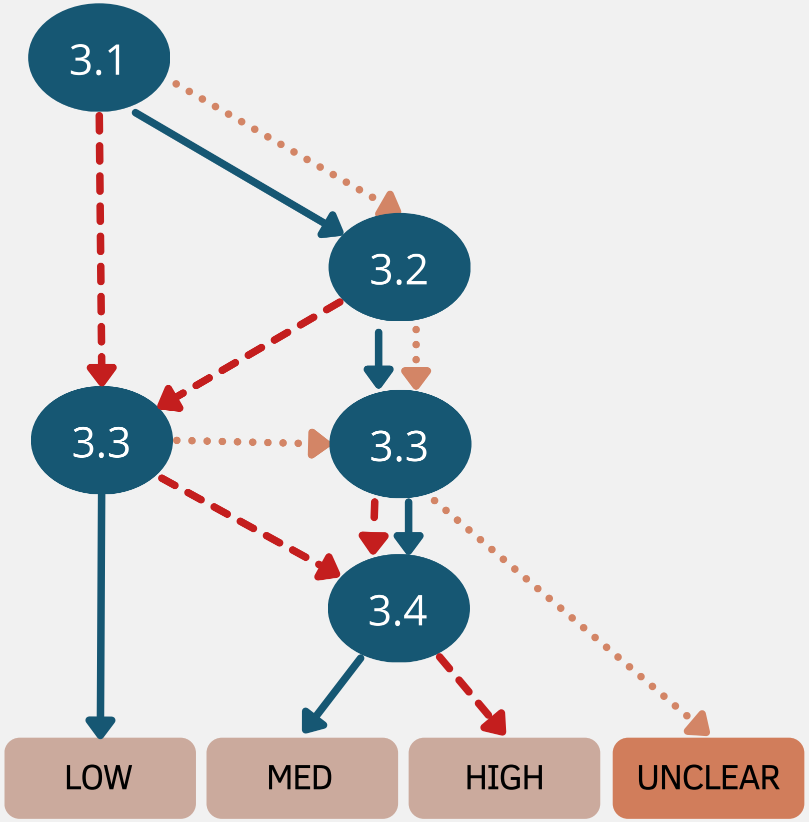

Criterion 4.
Risk of measurement biases

4.1 Can the awareness of the study question and design bias the measurement of the response variable (= GHG emissions) (un-)intentionally?

4.2 Was the response variable (= GHG emissions) measured appropriately to assess the impact of land use?

4.3 Were the methods for measuring GHG emissions the same across groups?

4.4 Were the potential differences in measured outcomes between groups adjusted?

RISK OF BIAS

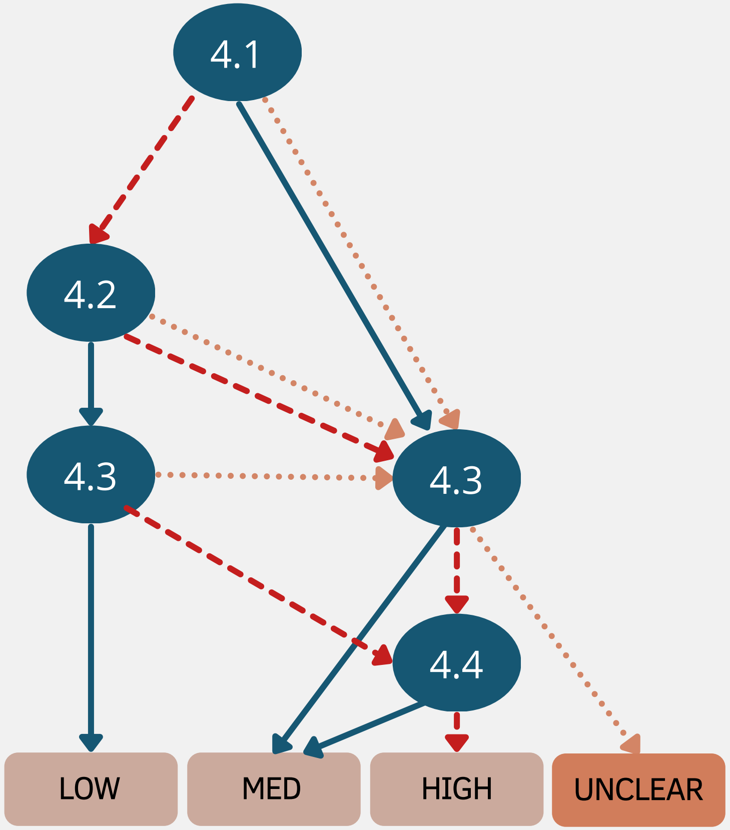

Criterion 5.
Risk of outcome assessment biases

5.0 Were inferential statistics used on the data being extraced?

5.1 Is it likely that there are errors in the applied descriptive statistical analyses?

5.2 Is it likely that there are errors in the applied inferential statistics (including null hypothesis testing, estimation, coding)?

5.3 Were statistical methods appropriate?

RISK OF BIAS

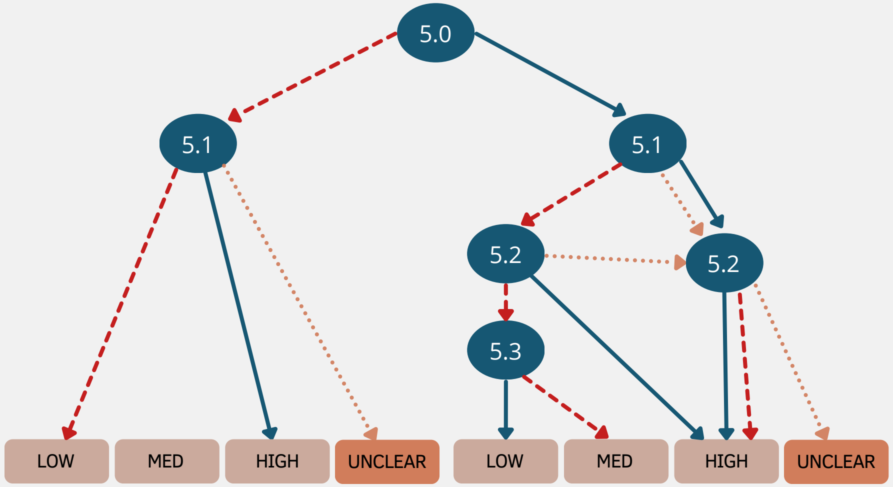

Criterion 5.
Risk of outcome assessment biases

5.0 Were inferential statistics used on the data being extraced?

5.1 Was the data analyst aware of the intervention (=land use) received by the study areas?

5.2 Is it likely that there are errors in the applied descriptive statistical analyses?

5.3 Is it likely that there are errors in the applied inferential statistics (including null hypothesis testing, estimation, coding)?

5.4 Were statistical methods appropriate?

RISK OF BIAS

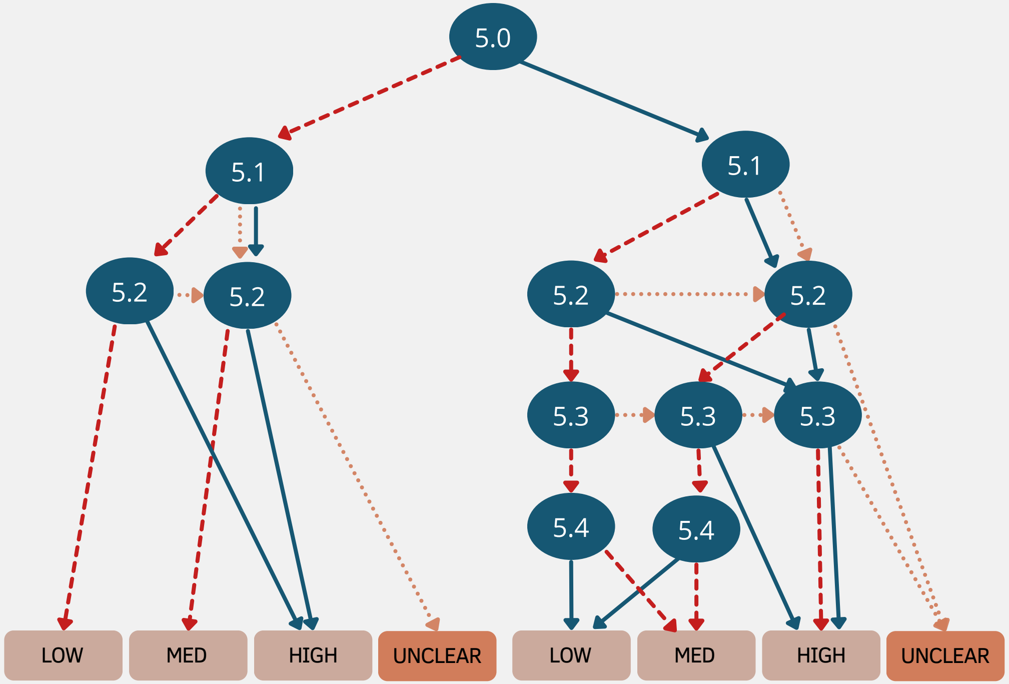

Supplement: Supplementary file 4 — Additional file 4. Critical appraisal tool visualisation. [file 13750_2023_310_MOESM4_ESM.pdf]
